# When and Where Birth Spacing Matters for Child Survival: An International Comparison Using the DHS

**DOI:** 10.1007/s13524-019-00798-y

**Published:** 2019-07-03

**Authors:** Joseph Molitoris, Kieron Barclay, Martin Kolk

**Affiliations:** 1 Centre for Economic Demography, Department of Economic History, Lund University, Lund, Sweden; 2 Hungarian Demographic Research Institute, Budapest, Hungary; 3 Max Planck Institute for Demographic Research, Rostock, Germany; 4 Department of Social Policy, London School of Economics and Political Science, London, UK; 5 Demography Unit, Department of Sociology, Stockholm University, Stockholm, Sweden; 6 Center for the Study of Cultural Evolution, Stockholm University, Stockholm, Sweden; 7 Institute for Future Studies, Stockholm, Sweden

**Keywords:** Birth spacing, Infant mortality, Developing countries, International comparison

## Abstract

A large body of research has found an association between short birth intervals and the risk of infant mortality in developing countries, but recent work on other perinatal outcomes from highly developed countries has called these claims into question, arguing that previous studies have failed to adequately control for unobserved heterogeneity. Our study addresses this issue by estimating within-family models on a sample of 4.5 million births from 77 countries at various levels of development. We show that after unobserved maternal heterogeneity is controlled for, intervals shorter than 36 months substantially increase the probability of infant death. However, the importance of birth intervals as a determinant of infant mortality varies inversely with maternal education and the strength of the relationship varies regionally. Finally, we demonstrate that the mortality-reducing effects of longer birth intervals are strong at low levels of development but decline steadily toward zero at higher levels of development. These findings offer a clear way to reconcile previous research showing that birth intervals are important for perinatal outcomes in low-income countries but are much less consequential in high-income settings.

**Electronic supplementary material:**

The online version of this article (10.1007/s13524-019-00798-y) contains supplementary material, which is available to authorized users.

## Introduction

The World Health Organization (WHO) has identified birth interval length (the period between two consecutive live births) as a critical determinant of child mortality risks, recommending that women space their births between three and five years apart to reduce health risks to children and mothers (WHO 2007). This recommendation is based on the findings that intervals shorter than 36 months and longer than 60 months are associated with an elevated risk of infant death and other adverse outcomes (Conde-Agudelo et al. 2012; Hobcraft et al. 1985; Rutstein 2005). The relationship between short birth intervals, in particular, and mortality has been remarkably consistent, having been demonstrated repeatedly in a variety of developmental contexts across time and space (Becher et al. 2004; Cleland and Sathar 1984; Curtis et al. 1993; Miller et al. 1992; Millman and Cooksey 1987; Nault et al. 1990; Palloni and Millman 1986; Pebley et al. 1991; Ronsmans 1996; Whitworth and Stephenson 2002). Despite the large body of literature supporting these long-standing conclusions, recent evidence from studies of other perinatal outcomes has called the importance of birth spacing for infant health into question (Ball et al. 2014; Class et al. 2017; Hanley et al. 2017). Identifying whether birth intervals are in fact an important determinant of perinatal outcomes requires confronting two significant shortcomings in the current body of literature: a failure to address potential estimation bias from unmeasured confounding, and a dearth of international comparisons.

Much of the previous literature on the relationship between birth intervals and infant mortality has not adequately addressed the issue of residual confounding by unobservable characteristics. Endogeneity is always a concern when studying the effects of fertility behavior on children’s outcomes (see, e.g., Angrist and Evans 1998; Angrist et al. 2010; Rosenzweig and Wolpin 1980), and this is no different when studying the effects of birth spacing. Unobserved maternal heterogeneity can easily bias estimates of fertility’s effects on child health. The importance of this issue has recently come to the fore: several studies of mothers in affluent countries have shown that after unobserved compositional differences between women are accounted for, birth intervals seem to be inconsequential for children’s perinatal outcomes, such as birth weight, the risk of preterm birth, and being small for gestational age (Ball et al. 2014; Class et al. 2017; Hanley et al. 2017). These research findings call into question whether birth intervals really matter for perinatal outcomes at all (Klebanoff 2017). At the same time, recent research on low-income populations has shown that even after unobserved maternal heterogeneity is adjusted for, birth intervals are still highly consequential for infant mortality in high-mortality populations, such as Bangladesh and sub-Saharan Africa (Kozuki and Walker 2013; Kravdal 2018; Molitoris 2018b).

Because the extant literature is largely composed of case studies, it has been difficult to determine the extent to which differences between findings have been due to methodologies, sample selection procedures, or contextual factors. The primary goal of this study is therefore to investigate how the relationship between preceding birth intervals (the duration of time between the births of the older preceding sibling and the index child) and infant mortality varies across developmental contexts while applying uniform methods that can minimize residual confounding from unobserved heterogeneity. The benefit of a standardized comparative approach is that it allows us to shed light on both the average effects of birth interval length on infant mortality and also whether the importance of birth intervals varies according to contextual conditions. An international comparison may help us to reconcile the apparently discrepant findings in the literature and provide benchmarks for knowing when increasing birth spacing may or may not be a relevant intervention for reducing infant mortality.

Our study addresses the aforementioned issues by using data from 77 countries and more than 200 waves of the Demographic and Health Surveys (DHS). First, we account for the probable endogenous relationship between birth spacing and infant mortality by estimating within-family linear probability models. These models can account for unobservable maternal factors, such as maternal health or shared frailty within the sibling group, which may be correlated with both interval length and infant mortality risks. Second, we explore how the relationship between birth intervals and infant mortality risks varies both within and between populations in order to identify whether specific groups of mothers drive any observed association. Finally, we link our estimates of birth intervals’ effects on infant mortality to several macro-level indicators of development in order to understand the conditions under which birth intervals are more or less important for child survival.

## Birth Intervals and Adverse Outcomes: Mechanisms and Findings

A detailed description of the theoretical mechanisms linking preceding birth intervals to children’s outcomes can be found elsewhere (Conde-Agudelo et al. 2012), but we briefly outline some of the leading explanations for why short birth intervals may be detrimental in some contexts but not in others. These mechanisms, which are not mutually exclusive, are maternal depletion, infection transmission, and sibling competition.

The maternal depletion hypothesis argues that shorter birth intervals do not allow women to fully physically recuperate from the previous pregnancy, which subsequently results in suboptimal fetal development and a higher risk of mortality for the child born following the short interval (Winkvist et al. 1992). In a context of chronic, continuous, and sustained foot shortages, a woman’s body prioritizes its own well-being over that of the fetus in distributing energy and nutrients (Ellison 2003; Peacock 1991). Such a physiological response is thought to preserve a woman’s potential for future reproduction as well as for lactation. While research continues to explore specifically what is depleted by one pregnancy and not sufficiently restored by the next (e.g., fat, micronutrients, muscle mass), some facts are well understood. For example, folate (vitamin B_9_), which is critical for the growth and development of the fetus and is generally replenished in the postnatal period, is less likely to return to optimal levels during shorter intervals (Greenberg et al. 2011).

Infection transmission is the second mechanism that may link birth intervals to infant mortality risks. The horizontal transmission hypothesis holds that closely spaced births will place the younger of the siblings at a greater risk of mortality (Boerma and Bicego 1992). The younger sibling will be exposed to a similar set of diseases as the older sibling while also having a less-developed immune system, which will increase the ease of transmission from the older to the younger sibling. The weaker immune system of the latter can also increase the lethality of infectious diseases. Some evidence indicates that for certain communicable childhood diseases, such as measles, secondary infections acquired by an index child from their older sibling tend to have significantly higher case fatality rates (Aaby et al. 1984, 1986; Garenne and Aaby 1990).

The final mechanism linking intervals to mortality is sibling competition, which implies that closely spaced children are more likely to compete for the same resources, such as parental time and investment. Generally, competition for most resources would not be so much a result of the interval length per se but rather a result of an increase in family size, leading to a decrease in parental attention and investment in the first years of life for the index child. However, direct competition for one critical resource—breastmilk—would be directly related to the length of a birth interval. Some evidence from low-income countries suggests that breastfeeding-pregnancy overlap is not uncommon (Boerma and Bicego 1992; Molitoris 2018a; Ramachandran 2002) and may result in a lower quality and quantity of breastmilk for the child born following the interval, leading to diminished neonatal growth (Marquis et al. 2002, 2003).

Our discussion thus far has centered on mechanisms that would explain why *shorter* preceding birth intervals may cause adverse perinatal outcomes. This focus has been intentional given that the literature on the topic has overwhelmingly shown that shorter intervals are associated with higher rates of mortality, stillbirth, low birth weight, and other poor outcomes. However, a smaller literature shows that *long* intervals (i.e., longer than 60 months) are also disproportionately associated with higher risks of adverse maternal outcomes, such as preeclampsia and eclampsia, which are known to be associated with fetal loss and preterm birth (Conde-Agudelo and Belizán 2000; Conde-Agudelo et al. 2006; Skjærven et al. 2002; Zhu et al. 1999). Why longer intervals would be detrimental has not yet been firmly established, but one explanation—maternal regression—is that the longer a woman goes without conceiving a subsequent child, the more her physiology (and consequently her perinatal outcomes) resembles that of a woman during her first pregnancy (Zhu et al. 1999). Nevertheless, it is important to recognize that the exposure to intervals beyond 60 months is much smaller than the exposure to intervals shorter than, say, 24 months. In developing countries, approximately 25 % of births occur within 24 months of the preceding birth, but only about 6 % of births occur after 60 months (Rutstein 2005). Short birth intervals therefore pose a considerably greater risk in most populations.

The literature has consistently found that short interbirth intervals are predictive of adverse infant outcomes, but this is not a universal finding. Some recent studies of high-income populations in Sweden, Canada, and Australia have found that when controlling for unobserved maternal heterogeneity via sibling fixed effects, short birth intervals did not lead to higher risks of low birth weight, being small for gestational age, or preterm birth (Ball et al. 2014; Class et al. 2017; Hanley et al. 2017), suggesting that the apparent relationship between interval length and children’s outcomes may be attributable to the nonrandom distribution of birth intervals across mothers. Nevertheless, other recent research on infant and child mortality using the same statistical approach has found quite different results. Two studies of poor, high-mortality populations—specifically, nineteenth century Stockholm, Sweden, and contemporary Bangladesh—have shown that shorter birth intervals increased the risk of neonatal, postneonatal, and child mortality (Molitoris 2017, 2018b). Furthermore, the latter two studies presented results that may explain the discrepancy in findings mentioned earlier. First, the effects of birth interval length on mortality risks decreased over time as the overall level of mortality declined in Sweden (Molitoris 2017). Second, even within a high-mortality context, the size of the effects of interval length on mortality varied inversely with the educational level of the mother in Bangladesh (Molitoris 2018b).

Taken together, all these findings may fit into the same picture. Given the mechanisms outlined earlier in this section, one should expect that as economic and epidemiological conditions improve, short birth intervals should become a less significant predictor of infant mortality. Maternal depletion, infection transmission, and resource competition should all become relatively less important as the general nutrition and health of the population improves, thereby making birth intervals a weaker determinant of infant mortality. To examine whether this is indeed the case, we apply uniform statistical methods that can account for unobserved heterogeneity to data from a variety of low- to middle-income contexts, and we explicitly examine whether the association varies across their respective levels of development.

## Data

### Demographic and Health Surveys

**Fig. 1:**
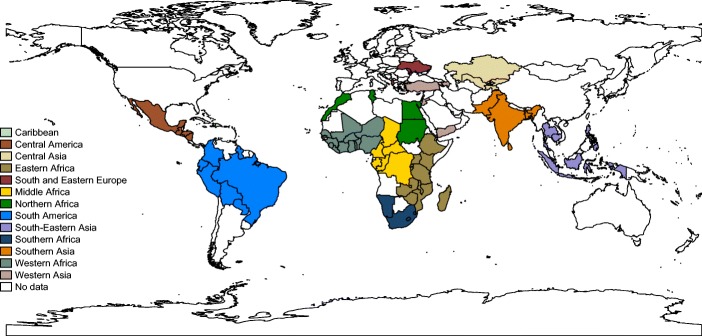
Map of countries included in analysis, grouped into UN subregions

This study used data on 77 countries and 207 waves of the Demographic and Health Surveys (DHS) (see Table A1 in the online appendix for list of included countries and their respective numbers of cases). The DHS is a household survey, with a separate survey for women aged 15–49. The household response rates in the surveys used in this study range from 83.8 % to 99.9 %, with a mean of 97.5 % and standard deviation of 2.45 %. The response rates for the woman’s questionnaire range from 77.0 % to 99.6 %, with a mean of 93.6 % and standard deviation of 3.92 %. Our analyses are based on the self-reported fertility histories of each woman surveyed. The outcome of interest in this study is infant mortality, defined as mortality between birth and 12 months. We restricted the pooled data in several ways for our analysis (see Fig. A1 in the online appendix for the sample selection flow chart). First, only children born at parities 2 or higher were included in the analysis because firstborns have an undefined preceding birth interval. Second, index children born as a set of a multiple birth (e.g., twin, triplet, and so on) were excluded. Third, children with very long birth intervals (greater than 10 years) were excluded from the analysis given that intervals of this length are highly unusual: 99 % of birth intervals are closed within 10 years in the data. Fourth, index children must have come from mothers with three or more births. This restriction was necessary because the within-family approach that we adopted requires at least two birth intervals (i.e., three births) per woman. In total, the final analytical sample included approximately 4.56 million births to more than 1.15 million women. Of these children, approximately 370,000 died in the first year of life. Figure 1 presents the geographical distribution of the countries included in our analysis.

**Fig. 2:**
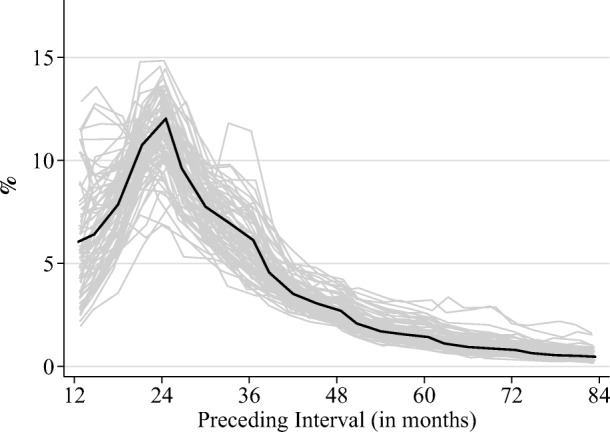
Distribution of preceding birth intervals (in months) in DHS countries. The bold line indicates the average distribution of all countries.

The distribution of birth intervals across the 77 countries is shown in Fig. 2. The mean birth interval was nearly 35 months (median = 29 months) with a standard deviation of 25 months. The distributions observed here followed typical distributions of birth spacing and were mostly similar across populations. Although the majority of populations conformed to the average distribution of intervals, some exceptional populations had unusually large shares of children born after very short birth intervals. For example, 15 % of children in Yemen were born following an interval less than 12 months, and for nearly one-quarter of the countries in our sample, more than 50 % of their birth intervals were less than 24 months. Such a high prevalence of short intervals is not necessarily indicative of data problems. Regional differences in spacing patterns across the developing world are pronounced (Casterline and Odden 2016). Nevertheless, our results rely heavily on the reliability of the birth histories. Therefore, to be certain that they are not biased due to misreporting of births, we also conducted several robustness checks, presented later in the article.

### World Bank Indicators of Development

To understand how the effects of birth intervals on infant mortality vary according to level of development, we linked our estimates to various indicators of development by country-year. We sourced the data from the World Development Indicators database, which is the primary World Bank collection of development indicators, compiled from officially recognized international sources. This database presents the most current and accurate global development data available, and it includes national, regional, and global estimates. Our analyses focused particularly on the national infant mortality rate (IMR) and total fertility rate because these were widely available across the countries and years in the DHS data.

## Methods

To analyze the effects of birth spacing on infant mortality, we estimated the following linear probability model:

1 $$ {Y}_{ij}={S}_{ij}{\upbeta}_{1, ij}+{\mathbf{X}}_{ij}{\upbeta}_{k, ij}+{\uptheta}_j+{\upvarepsilon}_{ij}. $$

The dependent variable, *Y*, is binary and indicates whether child *i* of mother *j* died in the first year of life. Our main independent variable, *S*, is the length of the preceding interbirth interval (i.e., the time between the birth of the older adjacent sibling and the birth of the index child). We treated it as a continuous variable with a quartic functional form in order to account for the possibility of a nonlinear relationship between interval length and mortality risks (Hobcraft et al. 1985; Rutstein 2005) (see Fig. A12 in the online appendix for evaluation of functional form). Because a major goal of this study is to provide comparable estimates across many populations, we adopted parsimonious models that control for basic demographic characteristics that may vary across siblings. The controls, **X**, include the sex of the index child, (centered) birth year, survival status of the previous child at the time of the index child’s birth, and birth order. Summary statistics of the model’s covariates may be found in Table 1.

**Table 1:** Distribution of index children’s selected characteristics

	*N*	%	IMR	Mean Interval	SD Interval
Infant Deaths	369,227	8.1			
Preceding Interval (in months)					
<12	147,128	3.2	210.1	0.84	0.08
12–14	265,978	5.8	156.5	1.09	0.07
15–17	280,031	6.1	124.6	1.34	0.07
18–20	344,059	7.5	104.7	1.59	0.07
21–23	474,945	10.4	92.9	1.84	0.07
24–26	533,548	11.7	81.5	2.08	0.07
27–29	423,655	9.3	75.5	2.33	0.07
30–32	338,117	7.4	67.5	2.58	0.07
33–35	301,998	6.6	59.5	2.83	0.07
36–38	265,019	5.8	53.3	3.08	0.07
39–41	195,262	4.3	51.7	3.33	0.07
42–44	149,155	3.3	50.4	3.58	0.07
45–47	128,310	2.8	44.9	3.83	0.07
48–50	112,670	2.5	41.1	4.08	0.07
51–53	86,132	1.9	41.0	4.33	0.07
54–56	69,504	1.5	40.9	4.58	0.07
57–59	62,972	1.4	38.7	4.83	0.07
60–62	57,423	1.3	37.6	5.08	0.07
63–65	44,476	1.0	38.6	5.33	0.07
66–68	37,336	0.8	37.2	5.58	0.07
69–71	34,062	0.8	36.9	5.83	0.07
72–74	31,416	0.7	36.3	6.08	0.07
75–77	25,078	0.6	36.6	6.33	0.07
78–80	20,942	0.5	36.5	6.58	0.07
81–83	19,617	0.4	38.2	6.83	0.07
84+	115,295	2.5	37.2	8.11	0.83
Sex					
Male	2,328,349	51.0	85.5	2.73	1.50
Female	2,235,779	49.0	76.1	2.74	1.50
Survival Status of Previously Born Sibling					
Alive	3,868,540	84.8	63.7	2.82	1.51
Died	695,588	15.2	176.5	2.27	1.32
Birth Order					
2	1,140,772	25.0	86.1	2.58	1.40
3	1,149,562	25.2	71.0	2.85	1.60
4	803,513	17.6	75.4	2.81	1.54
5	548,504	12.0	80.4	2.78	1.50
6	368,638	8.1	85.2	2.73	1.46
7	239,333	5.2	90.2	2.70	1.42
8+	313,806	6.9	100.9	2.60	1.35
Maternal Education					
No education	2,094,677	45.9	101.1	2.63	1.36
Primary	1,635,935	35.9	73.2	2.75	1.52
Secondary	713,587	15.6	47.2	2.93	1.70
Tertiary	118,939	2.61	32.1	3.08	1.87
Missing/unknown	990	0.02	82.8	2.66	1.47
UN Subregion					
Caribbean	166,187	3.6	61.55	2.64	1.57
Central America	155,316	3.4	57.66	2.64	1.52
Central Asia	36,549	0.8	56.58	2.87	1.73
Eastern Africa	782,653	17.2	88.55	2.73	1.37
Middle Africa	208,658	4.6	79.34	2.76	1.42
Northern Africa	289,526	6.3	81.02	2.66	1.55
South America	533,459	11.7	68.58	2.83	1.73
Southeastern Asia	502,336	11.0	72.75	2.88	1.68
Southern Africa	66,759	1.5	61.15	3.26	1.76
Southern and Eastern Europe	689,361	15.1	85.10	2.62	1.39
Southern Asia	8,948	0.2	44.14	3.22	1.85
Western Africa	939,886	20.6	98.16	2.75	1.35
Western Asia	184,490	4.0	54.91	2.41	1.47
	*N*	Mean	SD	Min.	Max.
Birth Year	4,564,128	1990.67	10.48	1952	2014
Preceding Interval (in years)	4,564,128	2.73	1.50	0.50	9.92

Most previous studies on this topic in low-income countries have not addressed the probable endogeneity of birth interval length when studying its effects on infant health. Interval length may be correlated with a host of characteristics that may be unobserved, such as maternal breastfeeding preferences or health behaviors, and may themselves influence the probability of infant mortality. Recent work has called attention to the importance of accounting for unobserved factors that may bias estimates of the effect of birth spacing on child outcomes (Ball et al. 2014; Barclay and Kolk 2017; DaVanzo et al. 2008; Kravdal 2018; Molitoris 2017, 2018b). We therefore partitioned the error term into a mother-specific component, θ, and an individual-specific component, ε, by subtracting the within-mother means of all variables from their observed values. This allowed us to estimate within-family models by controlling for sibling fixed effects (FE). Thus, our models compared children born to the same mother. Our results therefore should not be driven by unobserved, time-invariant differences between mothers that correlate with interval length, such as religious affiliation; ever-born number of children; age at first birth; ethnicity; country and survey effects; or, insofar as it is time-invariant, socioeconomic status, among other factors. Sibling FE also allowed us to control for the shared propensity for infant mortality within a given family (i.e., shared frailty). Recent work has compared cousins for the same reasons mentioned earlier (Class et al. 2017), but this was not feasible in the present study because cousins cannot be identified in the DHS data.

The within-family approach is not without limitations, however. First, we were unable to control for any source of endogeneity that emerges as a result of *time-varying* unobserved heterogeneity that is not captured by birth order or birth cohort. With that in mind, our modelling strategy does, however, offer a more robust control strategy than has generally been applied. Second, the within-family approach necessarily restricted our analysis sample to only women with three or more births. However, because we are studying high-fertility populations, the problem this restriction poses for the generalizability of the findings is not severe. Nearly 77 % of all children in the DHS come from family sizes of three or more. Because we are interested in only higher-order births, our analysis actually captures the vast majority of infants who could be affected by birth spacing: one-child sibling groups do not contribute any observations to the universe of birth intervals, and two-child sibling groups contribute only one birth interval. In contrast, a three-child sibling group contributes twice as many birth intervals to the universe of birth intervals as a two-child group, a four-child group contributes three times as many, and so on. Given the high fertility in our data, we calculated that our focus on sibling groups with at least three children includes 91.5 % of the measurable birth intervals in the surveys. Finally, a within-family analysis will also disproportionately exclude more recent maternal cohorts (with respect to the interview date) who have not yet had three or more children, although it will not exclude women who gave birth at younger ages in older cohorts.

In our analysis, we first compared the between-family estimates (ordinary least squares (OLS)) with the within-family estimates (FE) using the pooled sample of surveys to identify whether the relationship between preceding interval length and infant mortality persists after minimizing residual confounding from maternal heterogeneity. We then stratified the sample by United Nations subregion (see Table A6 in the online appendix for grouping) and maternal education to identify whether the relationship varies between or within populations. This exercise is valuable because it can highlight whether the aggregated patterns are being driven by a few exceptional parts of the world and can reveal whether infant mortality is more strongly linked to birth spacing in some groups than others. Based on the theoretical mechanisms described previously, we would expect that children born to women with less education would be more vulnerable to infection or resource scarcity than those born to more highly educated women. Recent evidence from Bangladesh has indeed shown this to be the case (Molitoris 2018b), and it is important to identify whether this finding is generalizable to the rest of the world. More precise targeting of vulnerable groups by family planning programs may be required in order to offset recent funding cuts to international aid organizations (Bingenheimer and Skuster 2017; Starrs 2017).

After estimating these models, we then adopted a comparative perspective. Once again using the within-family approach, we estimated the association between birth intervals and mortality for each country-cohort combination in the pooled DHS sample. Because country does not vary between siblings and therefore cannot be included as a covariate in the model, we estimated separate models for each country and included an interaction term between the preceding birth interval and the birth year of the index child. We then estimated the effect of increasing the interval from 12 to 24 months on infant mortality for each birth cohort with at least 30 observations in each country-cohort combination. Next, we linked the estimates to World Bank data to examine whether the effects of birth intervals vary according to the level of development, proxied using data on the IMR and total fertility rate (TFR) for each country-birth cohort combination. These two indicators were chosen because they serve as good general proxies for social and economic development, and information on these indicators was also consistently available across countries and years.

## Results

### Controlling for Unobserved Heterogeneity

**Fig. 3:**
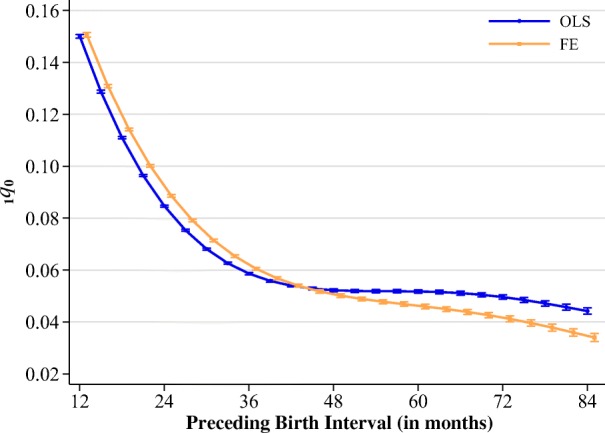
Probability of dying before age 1 at different preceding birth interval lengths predicted by OLS and FE models. Error bars represent 95 % confidence intervals.

To begin our analysis, we first estimated the model described in Eq. (1) with and without controls for sibling FE. To facilitate the discussion of the results, we present the results graphically as predicted probabilities, but the full output of the models is available in the online appendix (Tables A2 and A3). Figure 3 shows the predicted probabilities of infant mortality by the length of the preceding birth interval for the between-family (OLS) and within-family (FE) models. We estimated the probabilities while holding all other variables at their means. Both the between- and within-family models provided fairly similar estimates on the effects of short birth intervals, pointing toward the same substantive conclusions: when intervals are shorter than about 24 months, increasing the length of the birth interval reduces the probability of infant mortality substantially. The only significant difference between the estimated effects emerged at longer birth intervals. The estimates from the between-family models suggest that the risk of infant mortality plateaus with intervals between 36 and 48 months in length. The within-family estimates, on the other hand, diverged at this point. They showed that the probability of infant mortality continued to decline as intervals became longer, albeit at a much slower pace. In other words, the marginal benefit of increasing a birth interval when the interval was already greater than about 36 months was fairly small, whereas increasing the length of an interval shorter than 36 months would be highly beneficial in terms of reducing infant mortality risks. It is worth highlighting here that in spite of the WHO recommendation for optimal spacing between three and five years, we found no evidence of an *increase* in mortality risks with increasing birth intervals, which is also consistent with another recent study using DHS data examining neonatal and under-5 mortality (Kozuki and Walker 2013).

### Identifying Regional and Socioeconomic Variation

**Fig. 4:**
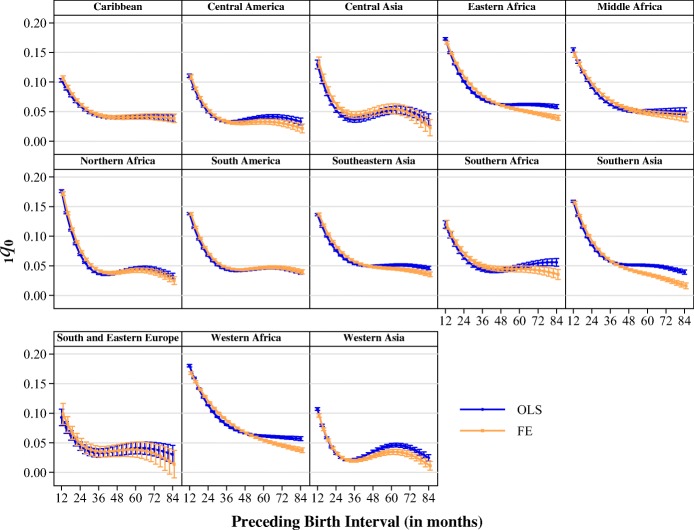
Predicted probabilities of dying before age 1 at different interval lengths and UN subregion. Estimates are from models stratified by UN subregion. A full list of countries included in regional groupings may be found in the online appendix. Error bars represent 95 % confidence intervals.

Next, we stratified the models according to 13 UN subregions and the mother’s highest level of education to explore heterogeneity in the relationship between birth intervals and infant mortality risks. Figure 4 shows the estimates from the models stratified by UN subregion. Regardless of region, birth intervals less than about 24 months were uniformly associated with a significantly higher risk of infant mortality. When we compared regions in terms of the percentage change in infant mortality associated with increasing birth intervals from 12 to 24 months in length, the smallest relative improvements in mortality were seen in the populations of Western, Middle, and Eastern Africa. In those populations, increasing birth intervals from 12 to 24 months was associated with about a 30 % reduction in infant mortality risks, with a more gradual decline in mortality risks with increasing intervals in those populations. On the other hand, the populations with the largest relative decrease in infant mortality for the same increase in spacing were those in Western and Central Asia, Northern Africa, and Central America, all of which showed an expected reduction of about 50 % when intervals increased from 12 to 24 months. In some regions, then, the optimal spacing for child survival appears to be considerably longer than in others. In regions such as South and Eastern Europe or the Americas, the benefits of increasing birth intervals beyond even 24 to 36 months seem negligible. Beyond intervals of that length, the mortality risk more or less plateaus. Yet in Eastern and Western Africa as well as Southern Asia, there appears to be a nearly linear negative relationship between birth interval length and mortality risks.

**Fig. 5:**
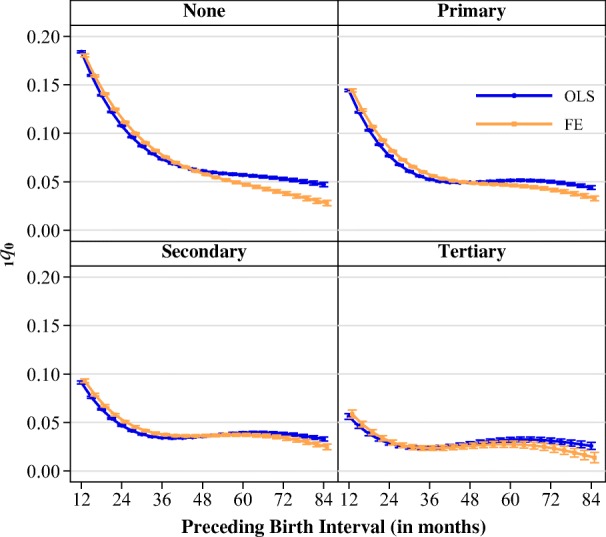
Predicted probabilities of dying before age 1 at different interval lengths and by mother’s educational attainment. Estimates are from models stratified by a woman’s highest level of education. Error bars represent 95 % confidence intervals.

Interestingly, the variation just described in the regional comparison also resembles the variation that we observed between educational groups (see Fig. 5). Among all women, children born after intervals shorter than 24 months had an elevated risk of infant mortality. Yet the magnitude of the mortality penalty for children born following shorter intervals varied inversely with a woman’s level of education. Children born to women with no education had a 0.18–0.07 probability of dying if they were born following an interval of 12 to 36 months. These probabilities declined as maternal education increased. Among women with a tertiary education, children born following the same interval lengths had a 0.06–0.02 probability of dying. As in the regional comparison, the point of diminishing returns to further spacing differed across educational groups. Women with no education showed the same pattern that characterized some of the least-developed regions: a nearly linear negative relationship between interval length and the probability of infant mortality. Children born to women with at least a primary education had a different pattern, in which the probability of dying declined until intervals reached about 36 months in length, after which the mortality risk plateaued. This pattern was also evident for women with secondary and tertiary education, with the only difference being the point at which mortality risks flattened out; at higher levels of education, the risks plateaued at shorter interval lengths. Note that stratifying our models by the education of the mother necessarily implies a change in the underlying populations being represented by each model, which may partially explain why the patterns of women with low education resemble those of the least-developed regions. For example, when we consider the group of women with tertiary education, they will be disproportionally drawn from more developed regions, where the relationship between spacing and mortality may be less dramatic. We addressed this issue by additionally estimating the stratified models for low-, medium-, and high-mortality contexts (see Fig. A2 in the online appendix). Here, each DHS survey was classified as low-mortality if the IMR was below 50 deaths per 1,000 live births, medium if it was between 50 and 100, and high if it was greater than 100. After we reestimated the models, the results were consistent with the patterns shown in Fig. 5. Regardless of the level of mortality in the particular survey, the educational gradient in the association between birth intervals and infant mortality remained. Within educational groups, the magnitude of the association was positively correlated with the level of infant mortality in the survey.

### Comparing the Effects of Spacing Across Levels of Development

**Fig. 6:**
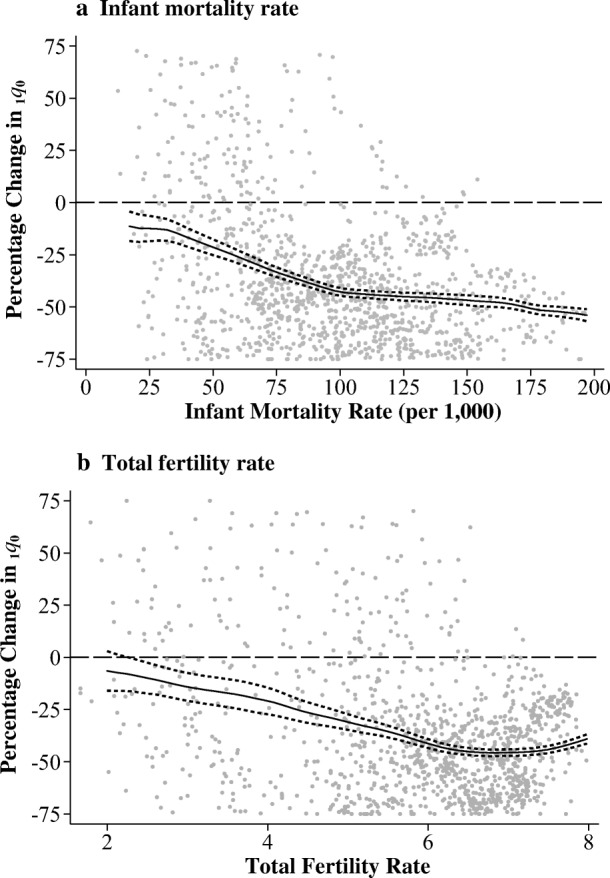
Marginal effect of increasing interval length from one to two years by the infant mortality rate (panel a) and total fertility rate (panel b). A kernel-weighted local polynomial smoothed trend was superimposed with 95 % confidence intervals.

The final part of our analysis compared the effects of spacing across levels of development. To do this, we estimated similar FE linear probability models as in Eq. (1) but included an interaction term between the length of the preceding birth interval and the birth year of the index child. We estimated these models separately for each country and then estimated the effect of increasing a birth interval from 12 to 24 months in each birth cohort of each country. This procedure effectively allowed us to generate more than 1,200 data points that can be plotted against the development indicators: the IMR and TFR (Fig. 6). The estimated effects were scaled to reflect a percentage change in the respective probabilities of dying before age 1 (_1*q*0_) in each country-cohort combination to allow for comparison across years and populations. The vertical axis can therefore be interpreted as the expected percentage change in the probability of dying before age 1 if a birth interval increased from 12 to 24 months in a specific country and cohort. All plots were fitted with a kernel-weighted local polynomial smoothed trend.

Panel a first plots the estimated effects against the national IMR. At levels of IMR greater than about 100 infant deaths per 1,000 live births, increasing a birth interval from 12 to 24 months was associated with a reduction in the probability of dying before age 1 by about 50 %, on average. Such large effects were persistent until the IMR fell well below 100, after which the protective effect of increasing a birth interval from 12 to 24 months began to weaken. At levels of IMR around 50 per 1,000 and lower, the effect of increasing intervals from 12 to 24 months clearly approached zero.

Panel b of Fig. 6 tells a very similar story. In high-fertility populations, the mortality reduction associated with increasing birth intervals was the largest. When national TFR was more than six births per woman, the marginal effect of increasing birth intervals from 12 to 24 months was about a 50 % reduction in the probability of dying. As fertility declines, we can see again that the marginal effect of spacing also declines. After the TFR fell below about six births per woman, the magnitude of the marginal effect decreased virtually linearly. When TFR fell below about three births per woman, the mortality-reducing effect of birth spacing was no longer statistically different from zero. Thus, panels a and b of Fig. 6 suggest that reducing the share of short birth intervals may be an effective measure for reducing infant mortality, but its potential benefits in both absolute and relative terms are stronger at lower levels of development. We also conducted the analysis with other indicators of development, such as the Human Development Index and life expectancy at birth, and the results pointed to the same substantive conclusions (see Fig. A3, online appendix).

### Supplementary Analyses

In addition to our main results, we conducted several supplementary analyses to further explore heterogeneity in our findings and to check the robustness of our results. First, we stratified the models by a woman’s number of children ever born (CEB) and by index children’s birth cohorts to identify how consistent our findings are in small versus large families and to explore whether the patterns observed until now were driven exclusively by older birth cohorts. Because women with higher fertility will disproportionately contribute to the number of birth intervals and infant deaths in a population, we stratified our models by CEB to assess whether our findings are also generalizable to women with relatively low fertility. We also restricted our analysis to a subsample of births that occurred within the 10 years preceding the survey in order to account for the possible displacement or omission of births from women’s self-reported birth histories (Potter 1977). The displacement of births has been shown to be about 2 % or less within that time frame in the DHS (Pullum and Becker 2014). We also estimated our models using two subsamples of the data: one that included only even-parity births, and one that included only odd-parity births from families of five or more. We examined these subsamples because our analysis included a control for the death of the preceding child, which in a within-family framework allows the death of a single child to contribute to the variance of both the dependent variable and independent variable. To be sure that this did not affect our results in unanticipated ways, we reestimated the models on a subsample of children whose deaths cannot themselves enter into to the estimation as both dependent and independent variables. The focus on families with five children or more was simply for comparability between the two subsamples, given that the within-family framework requires at least two observations per family with a defined preceding interval and that and the first odd-numbered parities meeting that criterion are parities 3 and 5. This restriction, however, necessarily limits the generalizability of the sensitivity analysis with regard to the entire population of higher-order births. Finally, we checked the robustness of our comparative results by estimating the effects of increasing a birth interval from 18 to 30 and 24 to 36 months on infant mortality risks.

When we stratified our models by a woman’s total CEB, the relationship between interval length and infant mortality was stronger in larger families (see online appendix Fig. A4). In families of all sizes, a negative relationship emerged between interval length and mortality risks, but the differences were smallest in three-child families. In all family sizes above three, when intervals were shorter than 24 months, significant improvements in mortality risks could be gained by increasing spacing. From 36 months and more, we again found diminishing returns to lengthening intervals further. In families with three CEB, mortality risks declined more or less linearly as intervals grew longer. That the effects were largest in high-fertility families is consistent with previous findings by Kozuki and Walker (2013), who also used DHS data to investigate the relationship between mortality and birth spacing and showed that the association between short intervals and neonatal and under-5 mortality was strongest among women with high fertility.

In relation to the theoretical mechanisms described earlier, we can speculate that the negative association between birth interval length and infant mortality may be amplified by cumulative maternal depletion accompanying high parity, the increased propensity for the transmission of infection between siblings, or the greater division of resources in a large family. Although we could not include two-child families in our within-family analysis for econometric reasons, we could expect that the association between short birth intervals and infant mortality risks in these two-child families may be similar but attenuated further in comparison with the patterns observed in three-child families. This expectation was confirmed when we compared between-family estimates for two-, three-, and four-child families: our estimates in all cases were very similar, but the patterns were less pronounced in the smaller family sizes (see Fig. A5, online appendix).

Stratifying the analysis by birth cohort generated results similar to those found in the main analysis (see Fig. A6, online appendix). Regardless of period of birth, we again found the characteristic pattern of high mortality following intervals shorter than 24 months. The difference between the cohorts was that the mortality risk for earlier-born cohorts declined virtually linearly at longer intervals, whereas the mortality risk in later-born cohorts plateaued at intervals of approximately 36 months.

We then estimated the models for our three subsamples: (1) index children born within 10 years preceding the survey, (2) children from five-child families or larger born at even parities, and (3) those born at odd parities (see Figs. A7, A8, and A9, respectively, in the online appendix). The estimates and substantive findings based on these subsamples were consistent with the main findings. The one difference that emerged in the subsample of children born within the 10 years preceding the survey was that we no longer found a continued decline in the probability of dying at the longest intervals. Instead, the mortality risk plateaued at intervals 48 months or longer.

Finally, turning to the comparative analysis, the substantive findings of the robustness checks were similar to the original analysis, despite some differences (see Figs. A10 and A11, online appendix). Although we found a substantial weakening of the marginal effect of increasing birth intervals from 18 to 30 months or 24 to 36 months across levels of IMR and TFR, this effect was not to the same extent as when it is increased from 12 to 24 months. Keep in mind, however, that all findings in this article suggest that the substantial changes in mortality risks due to changing birth interval lengths have been almost exclusively driven by intervals less than two years in length. In other words, the main mortality-reducing effect of increasing birth intervals applies to those children born less than two years after their older sibling; and based on the previously discussed mechanisms, it is the effect of short intervals specifically that should be expected to change according to the context.

## Discussion

Our study produced several important findings. First, we showed that the relationship between birth interval length and infant mortality in low- and middle-income countries persists even after a within-family methodology is applied to account for unobserved heterogeneity between mothers. We found that the probability of dying is much higher at intervals below 24 months, and this pattern was highly consistent across regions of the world. Second, we found no evidence that intervals longer than 60 months are associated with an elevated probability of dying. On the contrary, the evidence presented here suggests that the probability of dying either plateaus or continues to decline, albeit at a slower pace, at longer birth intervals. Finally, and most significantly, the results from our international comparison showed that the importance of birth spacing as a determinant of infant mortality declines at more advanced levels of development. These findings have a number of important implications.

First, in contrast to recent studies using the same approach to analyze perinatal outcomes in populations from high-income countries, our study found that birth spacing does indeed have significant implications for infant survival, especially when intervals are shorter than 24 months. Because we adopted a within-family design, this pattern cannot be explained by unobserved heterogeneity between mothers.

Second, our results only partially support the WHO recommendation for spacing births between three and five years apart. The largest improvements in the probability of survival consistently come from increasing spacing until at least 36 months. Where our findings differ from the current recommendation is that we found little evidence that *longer* birth intervals will be detrimental for infant mortality. In most of our analyses, the probability of infant mortality plateaued at intervals of 36 to 48 months or even continued to decline at intervals longer than 48 months. In some of the UN subregions, we found evidence of a reversal in mortality risks followed by a continued decline, but these increases were often statistically indistinguishable from zero or were so slight as to be of little practical significance. Thus, although our results certainly support the idea of diminishing returns to longer spacing for mitigating infant mortality risks, they do not consistently support any upper bound for safe spacing. This finding is in line with recent work on low-income countries that has come to the same conclusion (Kozuki and Walker 2013), suggesting that guidelines for optimal spacing may need to be revised.

Third, in our international comparison, we showed that as the level of development increases, as measured by the level of infant mortality and total fertility, the average beneficial effect of increasing a birth interval from 12 to 24 months approaches zero. This finding was entirely consistent with the variation that we observed within populations, which showed that birth intervals were less consequential for infant mortality at higher levels of maternal education.

Finally, because we showed that the strength of the relationship between birth interval length and infant mortality declines as mortality and fertility fall, the comparative results here help to reconcile the differences in findings reported elsewhere. Recent research using data from high-income populations with low mortality and fertility cast doubt on the importance of interpregnancy intervals for poor perinatal outcomes, such as preterm birth and low birth weight (Ball et al. 2014; Class et al. 2017; Hanley et al. 2017). These studies also applied the same sibling FE approach used in this study in order to account for unobserved maternal heterogeneity. Consequently, it was unclear whether the discrepant findings in those studies were due to differences in methodologies, data, or context. Based on our comparative findings, it seems to be the latter. The null results from high-income contexts are entirely consistent with the patterns observed in low-income contexts. As development progresses, birth intervals become less significant for child health. Considering the causal mechanisms involved with this relationship, it would indeed be a surprise to find that birth intervals are significant for infant survival in contexts where infant mortality is extremely rare. In such populations, the average level of nutrition is high, and the burden of infectious diseases is low. Furthermore, the wide availability of both antenatal and postnatal medical interventions can save many vulnerable young lives. In low-income populations, however, where childhood stunting and wasting may be common, infectious disease is prevalent, family sizes are larger, and access to any modern medical care may be limited, infant mortality may be more sensitive to all inputs, including factors such as birth spacing. An additional implication of this finding is that it underscores the importance of promoting exclusive breastfeeding, especially in high-mortality populations. Breastfeeding has many known benefits, one of which is its ability to inhibit conception when practiced exclusively for up to six months (Kennedy et al. 1989). The continued promotion of exclusive breastfeeding could both directly reduce the risk of infant mortality by providing infants with optimal nutrition and indirectly reduce the risk of infant mortality by shifting the distribution of birth intervals in a population away from shorter intervals. This may be especially important for the populations of Central Africa, where recent declines in the durations of breastfeeding and postpartum abstinence have been responsible for stalls or reversals in respective fertility transitions (Rogers and Stephenson 2018).

Our study does have limitations to consider. First, we were able to consider the effects of only birth intervals, not interpregnancy intervals (i.e., the duration from the birth of one child to the conception of the next), on the risk of infant mortality. In our view, defining birth spacing in terms of the interpregnancy interval has two advantages: (1) it may provide a slightly better representation of women’s recuperative potential, and (2) it may also help to avoid misattributing the effects of preterm birth to those of short birth intervals. Nevertheless, the measure certainly would have drawbacks if it is self-reported, as it is in the DHS. If systematic differences exist in the misreporting of pregnancy durations or miscarriages, this would introduce greater uncertainty into our main exposure of interest. Furthermore, although the DHS includes information on time and length of pregnancies for a subset of children listed in the birth histories (usually the most recent pregnancy within the five years preceding the survey), its use would exclude many cases from our analysis, and its reliability is less clear. In addition, its use would eliminate the possibility of controlling for sibling FE, which was a central goal of this study. Second, because our estimates are based on within-family models, we were able to show that the relationship between birth intervals and infant mortality in low-income contexts is not attributable to time-invariant compositional differences between women. Nevertheless, our approach cannot remove the influence of within-family *time-varying* unobserved heterogeneity that is not captured by birth order or birth year and can be correlated with both interval length and infant mortality risks. Examples of such factors might include negative shocks to maternal health or socioeconomic resources in the household that could be correlated with infant mortality and reproductive behavior. In addition, the DHS data are based on self-reported fertility histories, which will undoubtedly introduce a certain degree of measurement error (Pullum and Becker 2014; Schoumaker 2014). However, we checked the sensitivity of our results to misreporting of births and found that the only difference in the findings appears to be that in the restricted sample, the estimated probability of dying plateaued after intervals longer than 48 months instead of continuing to decline. Finally, the development indicators that we drew from the World Bank refer to the national level of infant mortality and total fertility and thus may not closely correspond to the local conditions that the respondents to the survey actually experienced.

Nevertheless, our study also has important strengths. To our knowledge, this study is the first to apply a methodology that can account for unobserved heterogeneity in a comparative framework to identify the effects of birth spacing on infant mortality. In doing so, we confirmed many of the findings of previous research while also uncovering new details that can help revise general recommendations for birth spacing practices. By adopting a comparative approach, our study helps to reconcile some of the supposed inconsistencies in the current body of literature.

The findings presented here also offer several promising paths for future research. First, future research ought to focus more explicitly on identifying the causal mechanisms connecting birth interval length to infant mortality. Although our study sought to identify whether the relationship between birth spacing and mortality holds when adopting a robust control strategy, it was beyond its scope to identify which mechanisms facilitate this relationship. To explicitly identify the relative importance of the mechanisms linking short intervals to infant mortality, longitudinal data that include detailed information on factors such as biomarkers, household spending, and medical care would be required. Second, future work should also consider potentially different associations between birth spacing and mortality occurring at various times during infancy (i.e., early neonatal, late neonatal, postneonatal). Because the DHS is based on retrospective birth histories, deaths occurring in the early neonatal period may be especially prone to misreporting, and it is for this reason that we focused our study on infant mortality as a whole. Third, more comparative work that also includes wealthier populations would help to fill in the gaps regarding why birth intervals seem to matter a great deal in low-income contexts but much less in high-income contexts. Finally, it would be worthwhile to investigate whether the relationships between birth spacing and other outcomes are similarly moderated by the level of population health or other development indicators. Further comparative research may therefore help us to understand the conditions under which birth intervals matter for child health.

## Electronic supplementary material

ESM 1 (PDF 1792 kb)
