# Supplementary material for: When and Where Birth Spacing Matters for Child Survival: An International Comparison Using the DHS
Source: Demography. 2019 Jul 3;56(4):1349–70. doi: 10.1007/s13524-019-00798-y (PMC6667399; doi:10.1007/s13524-019-00798-y)
Supplement: Supplementary file 1 — (PDF 1792 kb) [file 13524_2019_798_MOESM1_ESM.pdf]

Online Appendix

When and Where Birth Spacing Matters for Child Survival: An International Comparison Using the DHS

Joseph Molitoris, Kieron Barclay, and Martin Kolk

Table A1. Country-specific samples included in final analysis.

Country	Waves	Survey Years	Women	Children	Deaths
Albania	1	2008-09	2,090	5,537	239
Armenia	2	2000; 2010	4,610	11,063	705
Azerbaijan	1	2006	2,267	5,895	502
Bangladesh	4	1993-94; 2004; 2007; 2011	32,372	117,981	11,012
Benin	4	2006; 2001; 1996; 2011-12	20,578	84,554	6,398
Bolivia	4	1989; 2003; 1994; 2008	24,423	102,322	10,389
Brazil	3	1986; 1991; 1996	7,810	30,344	3,083
Burkina Faso	4	1998-99; 2003; 1993; 2010	20,394	90,893	9,102
Burundi	2	1987; 2010	5,502	24,042	2,175
Cambodia	2	2000; 2005	18,965	73,996	6,951
Cameroon	4	2011; 1998; 2004; 1991	13,625	58,478	4,581
Central African Republic	1	1994-95	2,429	10,366	915
Chad	2	1996-97; 2004	6,678	31,008	3,626
Colombia	5	1995; 1990; 2000; 1986; 2010	34,775	112,410	4,074
Comoros	2	2012; 1996	2,710	11,795	748
Congo Brazzaville	2	2005; 2011-12	7,125	26,962	1,589
Cote D'Ivoire	2	1994; 2011-12	8,530	36,090	3,121
DR Congo	2	2007; 2013-14	12,539	54,897	4,507
Dominican Republic	6	1986; 2013; 2002; 1996; 1991; 2007	31,976	105,802	5,390
Ecuador	1	1987	1,776	7,348	694
Egypt	6	1992; 1995; 1988; 2008; 2014; 2000	56,254	208,862	16,910
El Salvador	1	1985	713	1,618	121
Ethiopia	2	2005; 2000; 2016; 2011	18,654	85,094	8,931
Gabon	2	2000; 2012	5,367	22,518	1,106
Gambia	1	2013	3,953	16,520	748
Ghana	5	2003; 1993; 2008; 1998; 1988	10,006	40,482	3,288

Guatemala	2	1995; 1987	8,148	36,547	3,195
Guinea	3	2012; 1999; 2005	11,191	47,818	5,425
Guyana	1	2009	1,753	6,083	235
Haiti	4	2005-06; 2000; 1994-95; 2012	13,217	55,962	4,660
Honduras	2	2005-06; 2011-12	15,179	58,138	1,989
India	3	2005-06; 1998-99; 1992-93	136,798	469,405	40,373
Indonesia	6	2007; 1994; 1987; 2002-03; 1991; 2012	84,009	296,467	22,846
Jordan	5	1990; 2002; 2007; 2012; 1997	25,272	114,643	3,532
Kazakhstan	1	1995	2,027	6,218	420
Kenya	5	1989; 1993; 2003; 1998; 2008-09	16,818	73,647	5,107
Kyrgyzstan	2	2012	4,271	13,380	735
Lesotho	2	2009; 2004	4,295	14,674	1,038
Liberia	3	1986; 2013; 2007	9,816	41,861	5,549
Madagascar	4	2003-04; 1997; 2008-09; 1992	15,931	68,670	5,435
Malawi	3	2010; 1992; 2000	23,952	98,928	10,011
Maldives	1	2009	3,006	11,855	588
Mali	5	1987; 2006; 2012-13; 1995-96; 2001	25,909	119,983	14,658
Mexico	1	1987	3,412	14,515	1,023
Moldova	1	2005	960	2,415	109
Morocco	3	2003-04; 1992; 1987	11,678	52,337	4,320
Mozambique	3	2003; 1997; 2011	14,806	59,647	6,824
Namibia	4	2006-07; 2000; 1992; 2013	9,627	34,763	1,931
Nicaragua	2	2001; 1998	10,487	44,498	2,627
Niger	4	2012; 1998; 2006; 1992	18,063	87,474	9,474
Nigeria	4	2003; 2008; 2013; 1990	39,157	174,953	17,302
Pakistan	3	2012-13; 2006-07; 1990-91	18,056	79,833	6,324
Paraguay	1	1990	2,123	9,496	408

Peru	6	1986; 1991-92; 2004-06; 2009; 1996; 2000	70,369	265,456	17,699
Philippines	5	1993; 1998; 2003; 2008; 2013	25,432	97,364	4,331
Rwanda	3	1992; 2010; 2000	16,331	71,932	6,912
Sao Tome & Principe	1	2008-09	1,147	4,429	230
Senegal	4	2010-11; 1992-93; 2005; 1986	27,206	121,012	8,963
Sierra Leone	2	2008; 2013	10,168	40,206	5,269
South Africa	1	1998	3,307	11,018	717
Sri Lanka	1	1987	2,954	10,287	371
Sudan	1	1989-90	3,474	17,516	1,375
Swaziland	1	2006-07	1,610	6,304	396
Tajikistan	1	2012	3,478	11,475	641
Tanzania	4	2010; 1991-92; 2004-05; 1996	17,761	77,778	6,723
Thailand	1	1987	2,775	9,398	511
Timor Leste	1	2009-10	5,555	25,111	1,904
Togo	3	2013-14; 1998; 1988	9,088	38,040	2,965
Trinidad & Tobago	1	1987	1,213	4,423	179
Tunisia	1	1988	2,537	10,811	851
Turkey	3	1993; 1998; 2003	9,003	32,347	2,923
Uganda	5	1995; 2011; 2000-01; 1988-89; 2006	16,980	78,993	6,986
Ukraine	1	2007	413	996	47
Uzbekistan	1	1996	1,604	5,476	272
Yemen	1	1991-92	3,851	20,542	2,468
Zambia	5	1992; 2007; 1996; 2001-02; 2013-14	19,949	87,004	7,042
Zimbabwe	5	1994; 2010-11; 1999; 1988; 2005-06	11,856	45,123	2,409
Total	207		1,154,143	4,564,128	369,227

Table A2. OLS and FE models of the effects of birth intervals on infant mortality.

	OLS		FE	
	B	*S.E.*	*B*	*S.E.*
Preceding Interval	-0.173	0.002	-0.153	0.002
Preceding Interval^2	0.048	0.001	0.040	0.001
Preceding Interval^3	-0.006	0.000	-0.005	0.000
Preceding Interval^4	0.000	0.000	0.000	0.000
Female	-0.009	0.000	-0.009	0.000
Birth year	-0.001	0.000	0.008	0.000
Birth year^2	0.000	0.000	0.000	0.000
Birth year^3	0.000	0.000	0.000	0.000
Previous Sibling Died	0.094	0.000	-0.072	0.000
Birth Order				
2	(ref)		(ref)	
3	-0.006	0.000	-0.033	0.000
4	-0.001	0.000	-0.063	0.001
5	0.003	0.000	-0.090	0.001
6	0.007	0.001	-0.115	0.001
7	0.012	0.001	-0.139	0.001
8+	0.020	0.001	-0.181	0.002
Constant	0.270	0.001		
Children	4,564,128		4,564,128	
Mothers	1,154,143		1,154,143	
F-statistic for model fit	11,867.1		5,171.8	
R^2	0.038		0.022	
F-statistic for FE			1.12	
Rho			0.322	

Note: Superscripts refer to higher-order polynomial terms.

Table A3. OLS and FE models of the effects of birth intervals on infant mortality using a categorical operationalization of interval length.

	OLS		FE	
	Coefficient	S.E.	Coefficient	S.E.
Preceding Interval				
<12	0.104	0.001	0.092	0.001
12-14	0.059	0.001	0.057	0.001
15-17	0.034	0.001	0.035	0.001
18-20	0.019	0.001	0.019	0.001
21-23	0.010	0.001	0.008	0.001
24-26	(ref)		(ref)	
27-29	-0.004	0.001	-0.004	0.001
30-32	-0.010	0.001	-0.011	0.001
33-35	-0.017	0.001	-0.021	0.001
36-38	-0.023	0.001	-0.028	0.001
39-41	-0.024	0.001	-0.029	0.001
42-44	-0.024	0.001	-0.028	0.001
45-47	-0.029	0.001	-0.035	0.001
48-50	-0.033	0.001	-0.039	0.001
51-53	-0.032	0.001	-0.038	0.001
54-56	-0.032	0.001	-0.037	0.001
57-59	-0.034	0.001	-0.043	0.001
60-62	-0.035	0.001	-0.045	0.001
63-65	-0.033	0.001	-0.044	0.002
66-68	-0.034	0.001	-0.043	0.002
69-71	-0.034	0.001	-0.046	0.002
72-74	-0.035	0.002	-0.048	0.002
75-77	-0.034	0.002	-0.046	0.002
78-80	-0.033	0.002	-0.048	0.002
81-83	-0.032	0.002	-0.048	0.002
84+	-0.032	0.001	-0.052	0.001
Female	-0.009	0.000	-0.009	0.000
Birth year	-0.001	0.000	0.008	0.000
Birth year^2	0.000	0.000	0.000	0.000
Birth year^3	0.000	0.000	0.000	0.000
Previous Sibling Died	0.094	0.000	-0.072	0.000
Birth Order				
2	(ref)		(ref)	
3	-0.006	0.000	-0.033	0.000
4	-0.002	0.000	-0.063	0.001

5	0.003	0.000	-0.090	0.001
6	0.007	0.001	-0.116	0.001
7	0.012	0.001	-0.140	0.001
8+	0.020	0.001	-0.181	0.002
Constant	0.070	0.000		
Children	4,564,128		4,564,128	
Mothers	1,154,143		1,154,143	
F-statistic for model fit	4982.69		2168.45	
R^2	0.038		0.022	
F-statistic for FE			1.12	
Rho			0.322	

Note: Superscripts refer to higher-order polynomial terms.

Table A4. OLS and FE models of the effects of birth intervals on infant mortality for children born within 10 years of the survey.

	OLS		FE	
	B	*S.E.*	*B*	*S.E.*
Preceding Interval	-0.167	0.002	-0.131	0.003
Preceding Interval^2	0.046	0.001	0.034	0.001
Preceding Interval^3	-0.005	0.000	-0.004	0.000
Preceding Interval^4	0.000	0.000	0.000	0.000
Female	-0.008	0.000	-0.008	0.000
Birth year	0.000	0.000	0.008	0.000
Birth year^2	0.000	0.000	0.000	0.000
Birth year^3	0.000	0.000	0.000	0.000
Previous Sibling Died	0.077	0.001	-0.169	0.001
Birth Order				
2	(ref)		(ref)	
3	-0.013	0.001	-0.046	0.001
4	-0.009	0.001	-0.086	0.001
5	-0.005	0.001	-0.122	0.002
6	0.000	0.001	-0.155	0.002
7	0.005	0.001	-0.187	0.002
8+	0.015	0.001	-0.230	0.003
Constant	0.273	0.002		
Children	2,329,949		2,329,949	
Mothers	922,402		922,402	
F-statistic for model fit	4,835.6		5,479.3	
R^2	0.030		0.055	
F-statistic for FE			1.12	
Rho			0.434	

Note: Superscripts refer to higher-order polynomial terms.

Table A5. OLS and FE models of the effects of birth intervals on infant mortality using a categorical operationalization of interval length for children born within 10 years of the survey.

	OLS		FE	
	Coefficient	S.E.	Coefficient	S.E.
Preceding Interval				
<12	0.101	0.001	0.078	0.001
12-14	0.058	0.001	0.048	0.001
15-17	0.034	0.001	0.031	0.001
18-20	0.020	0.001	0.017	0.001
21-23	0.010	0.001	0.007	0.001
24-26	(ref)		(ref)	
27-29	-0.006	0.001	-0.007	0.001
30-32	-0.012	0.001	-0.013	0.001
33-35	-0.019	0.001	-0.022	0.001
36-38	-0.022	0.001	-0.026	0.001
39-41	-0.023	0.001	-0.027	0.001
42-44	-0.024	0.001	-0.027	0.001
45-47	-0.028	0.001	-0.033	0.001
48-50	-0.030	0.001	-0.036	0.001
51-53	-0.030	0.001	-0.033	0.002
54-56	-0.030	0.001	-0.032	0.002
57-59	-0.030	0.001	-0.035	0.002
60-62	-0.032	0.001	-0.039	0.002
63-65	-0.031	0.002	-0.039	0.002
66-68	-0.030	0.002	-0.036	0.002
69-71	-0.031	0.002	-0.036	0.003
72-74	-0.032	0.002	-0.038	0.003
75-77	-0.031	0.002	-0.039	0.003
78-80	-0.029	0.002	-0.034	0.003
81-83	-0.028	0.002	-0.037	0.004
84+	-0.028	0.001	-0.036	0.002
Female	-0.008	0.000	-0.008	0.000
Birth year	0.000	0.000	0.008	0.000
Birth year^2	0.000	0.000	0.000	0.000
Birth year^3	0.000	0.000	0.000	0.000
Previous Sibling Died	0.077	0.001	-0.169	0.001
Birth Order				
2	(ref)		(ref)	
3	-0.013	0.001	-0.046	0.001
4	-0.010	0.001	-0.086	0.001

5	-0.005	0.001	-0.122	0.002
6	0.000	0.001	-0.155	0.002
7	0.005	0.001	-0.187	0.002
8+	0.014	0.001	-0.230	0.003
Constant	0.079	0.001		
Children	2,329,949		2,329,949	
Mothers	922,402		922,402	
F-statistic for model fit	2027.8		2285.44	
R^2	0.030		0.055	
F-statistic for FE			1.12	
Rho			0.434	

Note: Superscripts refer to higher-order polynomial terms.

Table A6. Countries included in UN sub-regions.

UN sub-region	Countries
Caribbean	Dominican Republic; Haiti; Trinidad & Tobago
Central America	El Salvador; Guatemala; Honduras; Mexico; Nicaragua
Central Asia	Kazakhstan; Kyrgyzstan; Tajikistan; Uzbekistan
Eastern Africa	Burundi; Comoros; Ethiopia; Kenya; Madagascar; Malawi; Mozambique; Rwanda; Tanzania; Uganda; Zambia; Zimbabwe
Middle Africa	Cameroon; Central African Republic; Chad; Congo Brazzaville; DR Congo; Gabon; Sao Tome & Principe
Northern Africa	Egypt; Morocco; Sudan; Tunisia
South America	Bolivia; Brazil; Colombia; Ecuador; Guyana; Paraguay; Peru
South-eastern Asia	Cambodia; Indonesia; Phillipines; Thailand; Timor Leste
Southern Africa	Lesotho; Namibia; South Africa; Swaziland
Southern and Eastern Europe	Bangladesh; India; Maldives; Pakistan; Sri Lanka
Southern Asia	Albania; Moldova; Ukraine
Western Africa	Benin; Burkina Faso; Cote DIvoire; Gambia; Ghana; Guinea; Liberia; Mali; Niger; Nigeria; Senegal; Sierra Leone; Togo
Western Asia	Armenia; Azerbaijan; Jordan; Turkey; Yemen

Note: Regional groupings may be found at https://unstats.un.org/unsd/methodology/m49/.

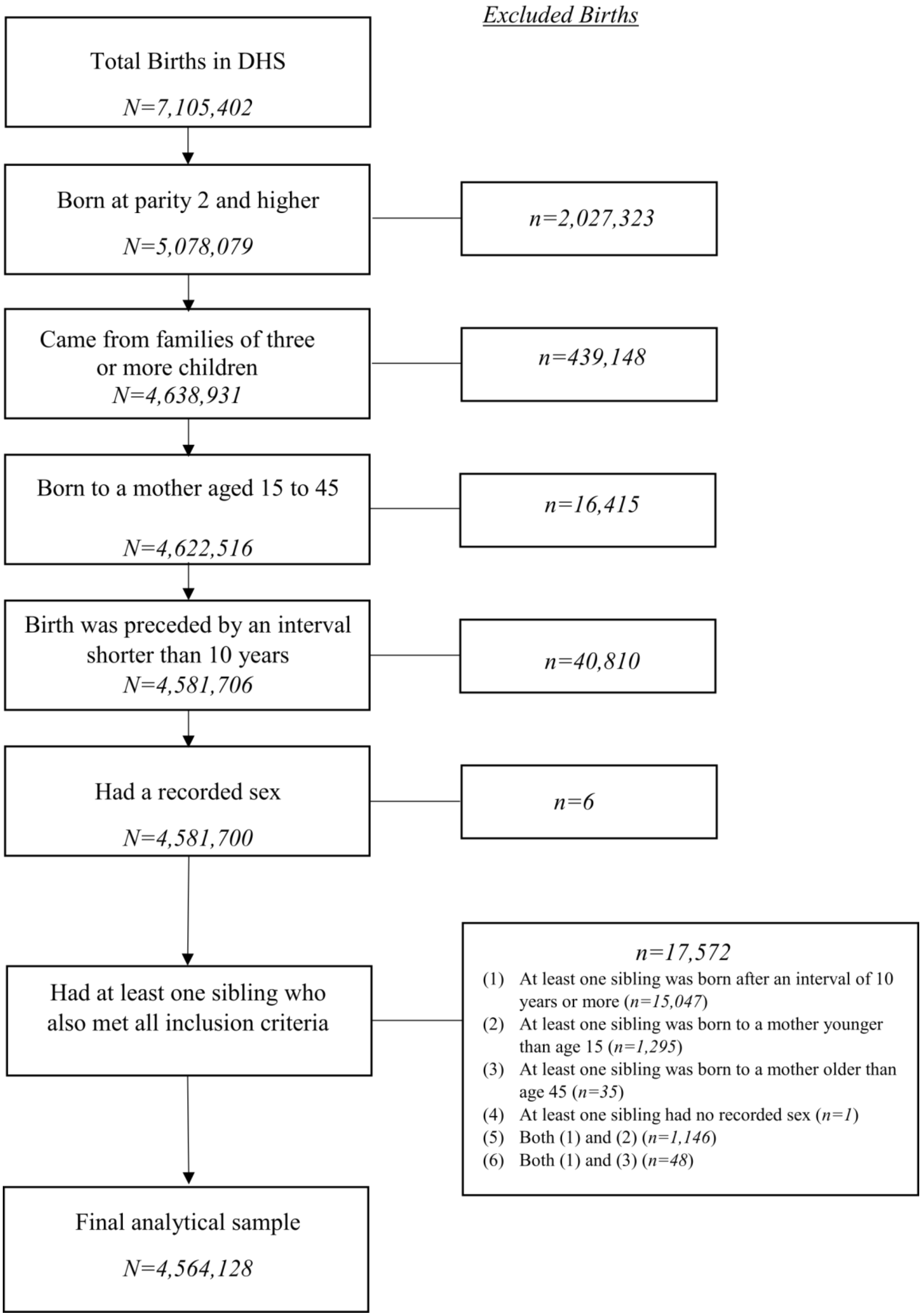

Figure A1. Sample selection flow chart.

I. STRATIFIED ANALYSES

(1) Low Mortality (IMR <50 per 1000)

(a) No Education

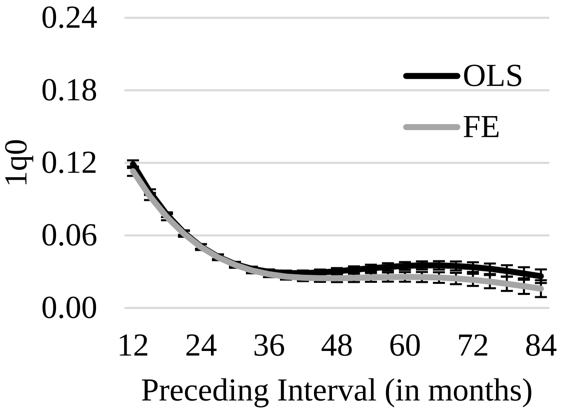

(b) Primary

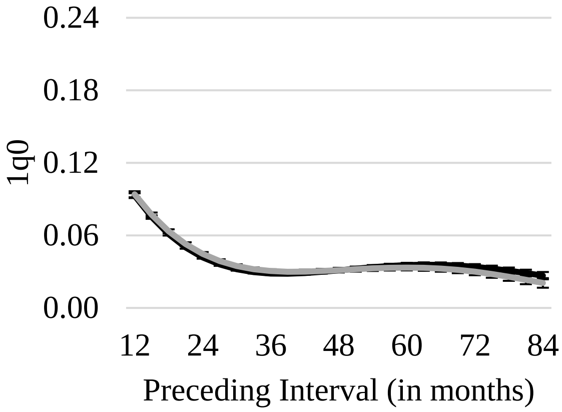

(c) Secondary

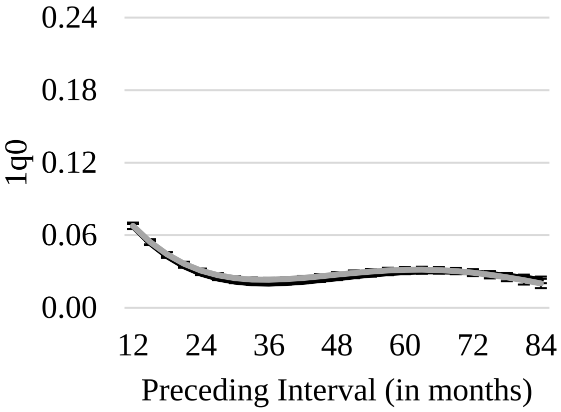

(d) Tertiary

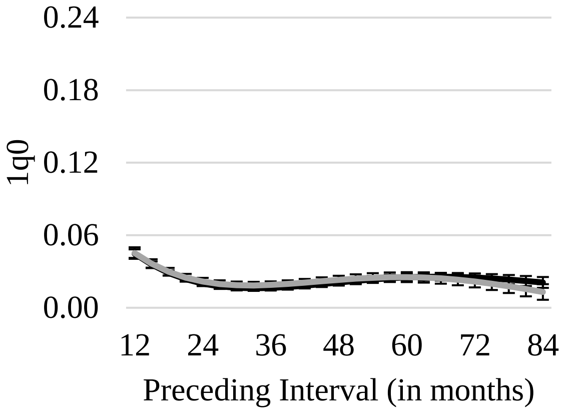

(2) Medium Mortality (100>IMR≥50)

(a) No Education

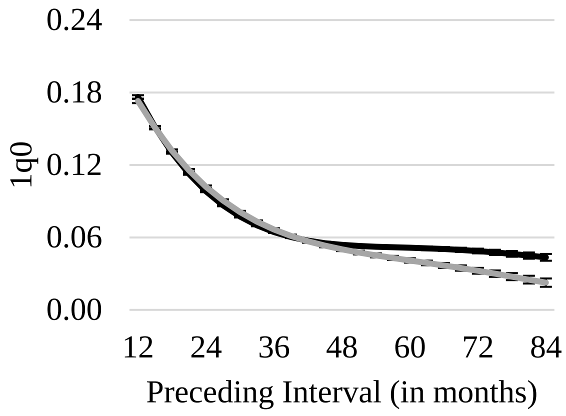

(b) Primary

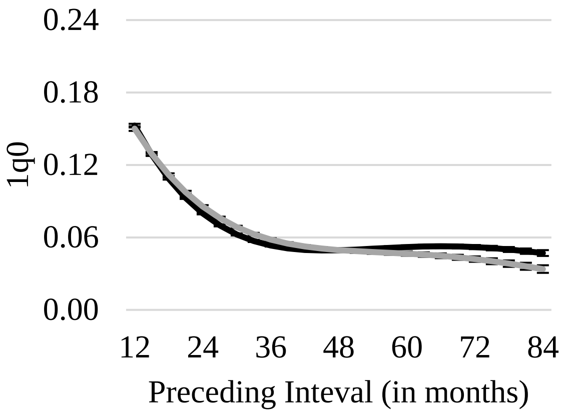

(c) Secondary

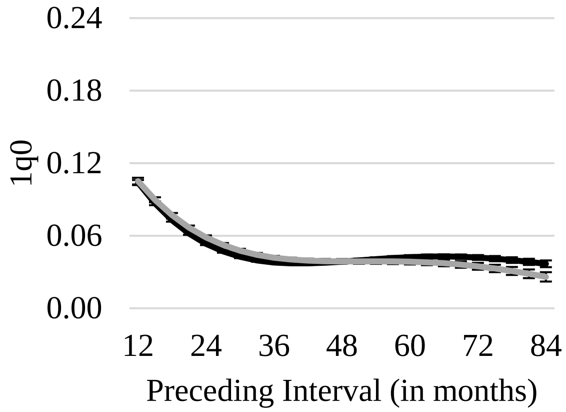

(d) Tertiary

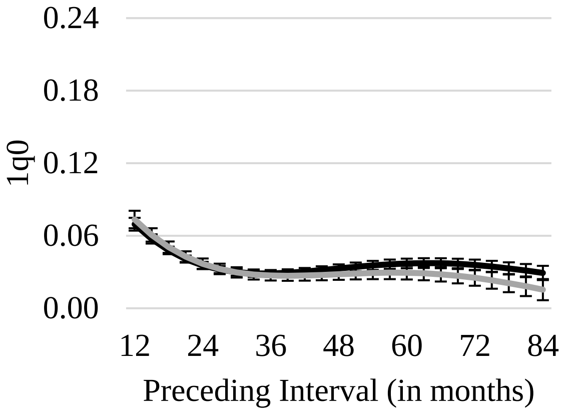

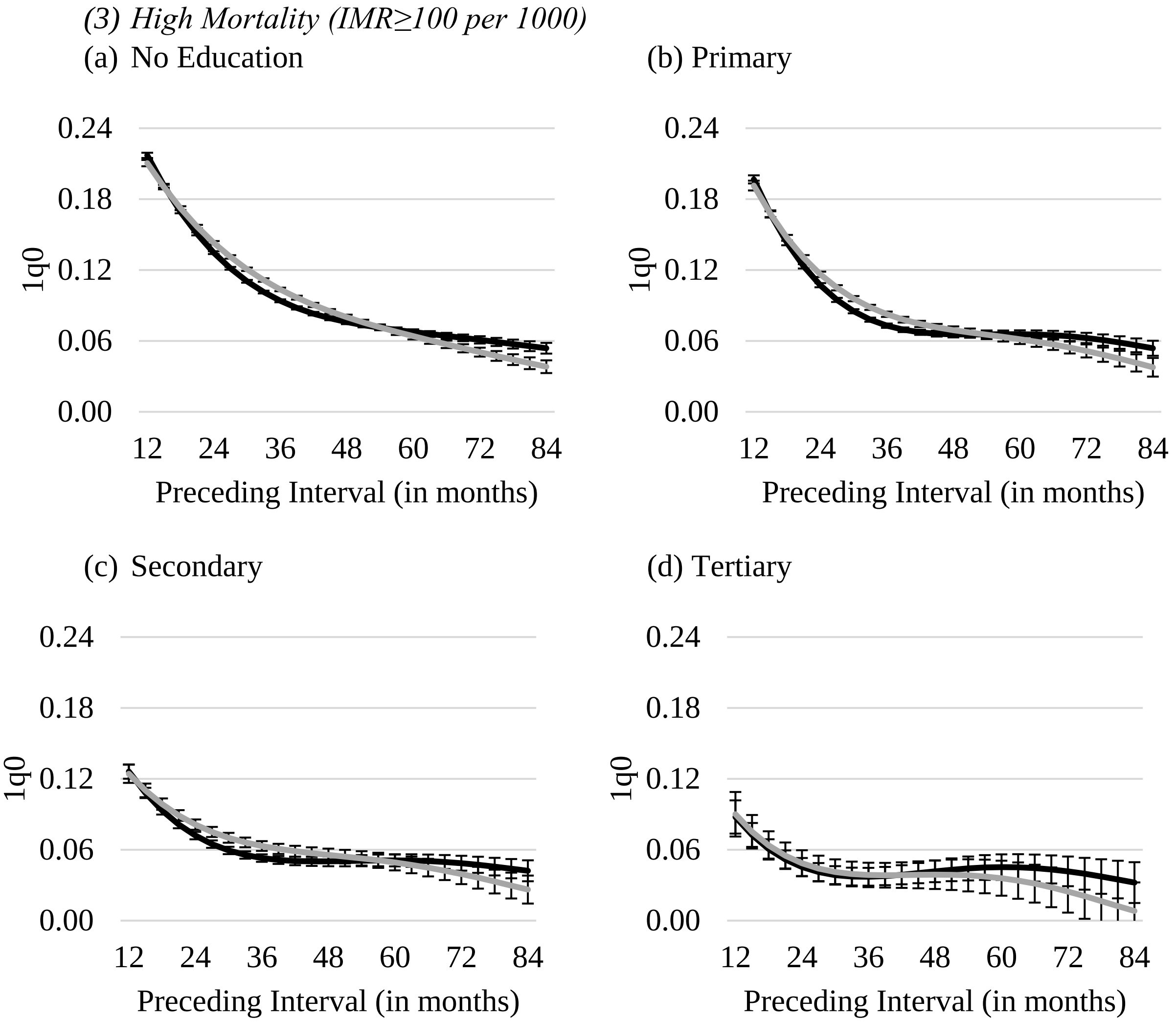

Figure A2. Predicted probabilities of dying before age one at different interval lengths and by mother's educational attainment by infant mortality context.

Note: Estimates are from models stratified by a woman's highest level of education and the overall level of infant mortality recorded for all births in a particular survey. Error bars represent 95% confidence intervals.

(a) Life expectancy at birth (both sexes) (b) Human Development Index

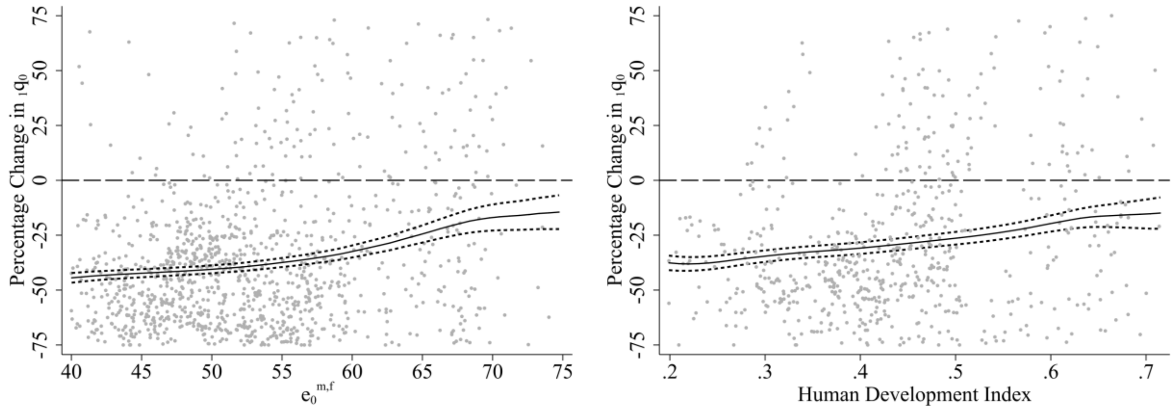

Figure A3. Marginal effect of increasing interval length from 12 to 24 months by the (a) life expectancy at birth and (b) Human Development Index.

Note: A kernel-weighted local polynomial smoothed trend has been superimposed with 95% confidence intervals.

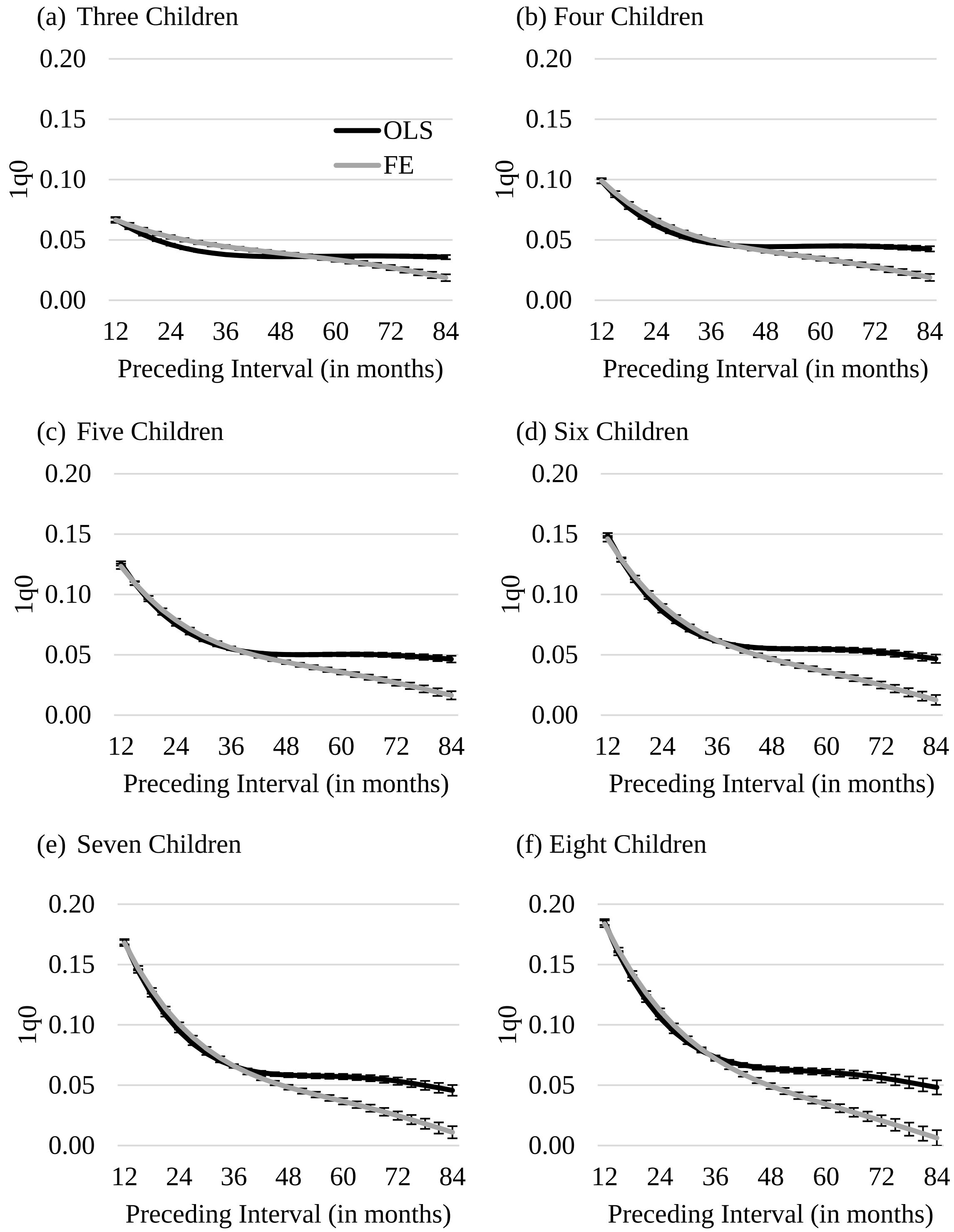

Figure A4. Predicted probabilities of dying before age one at different interval lengths and by total children ever born at the time of interview.

Note: Estimates are from models stratified by a mother's number of children ever born. Error bars represent 95% confidence intervals.

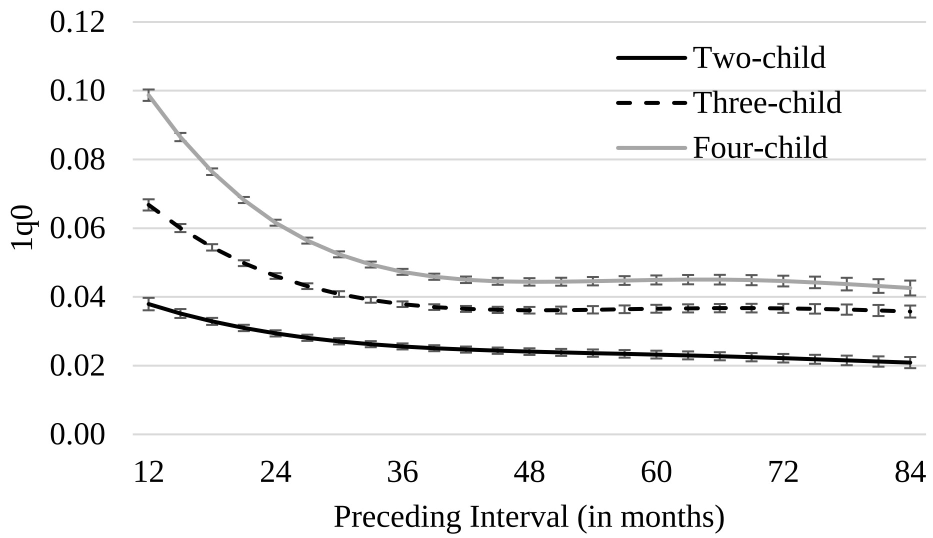

Figure A5. Comparison of predicted probabilities of dying before age one by interval length from between-family models for two-child, three-child, and four-child families.

Note: Error bars represent 95% confidence intervals.

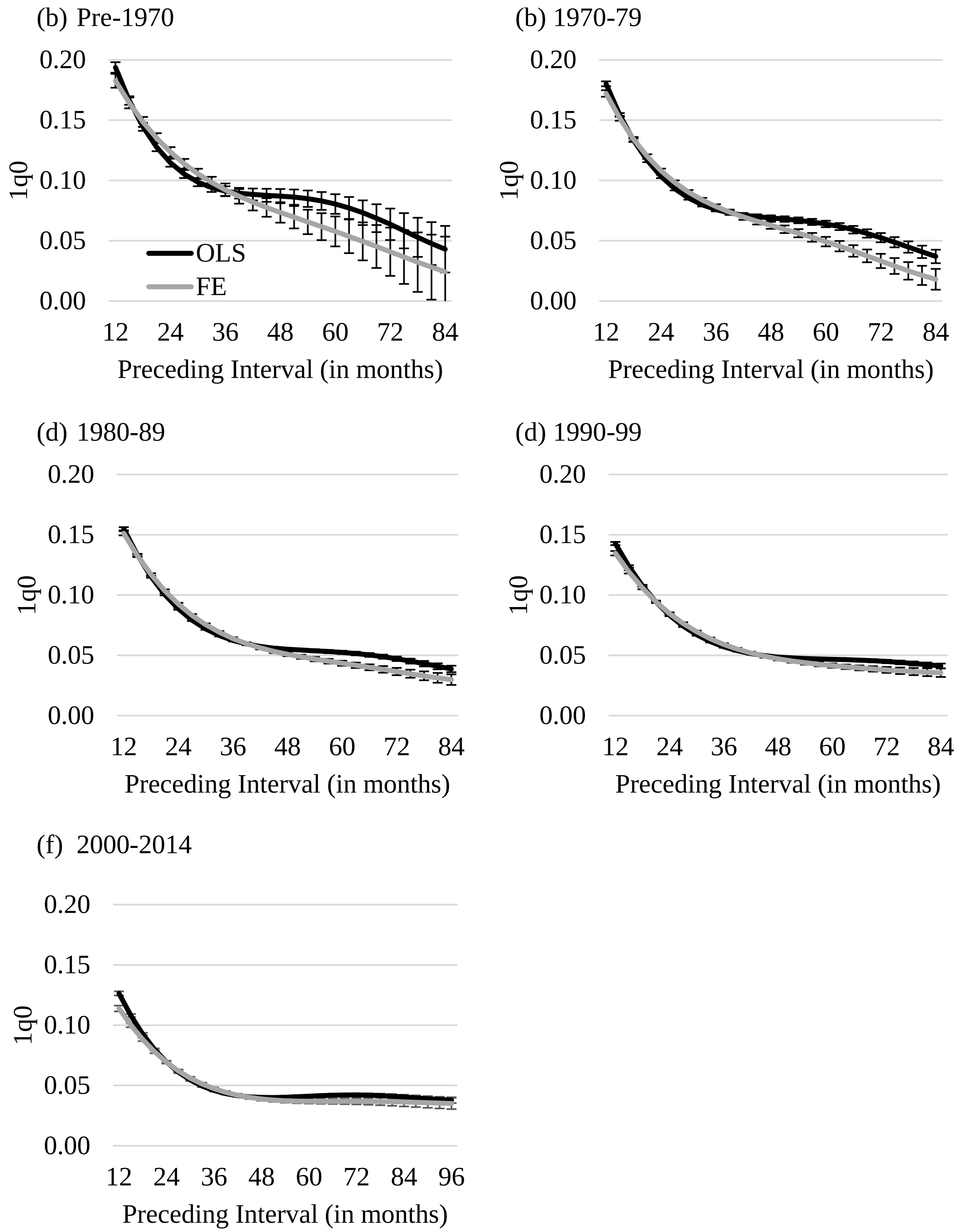

Figure A6. Predicted probabilities of dying before age one at different interval lengths and by index child's birth cohort.

Note: Estimates are from models stratified by the index child's birth cohort. Error bars represent 95% confidence intervals.

II. Sub-Sample Analyses

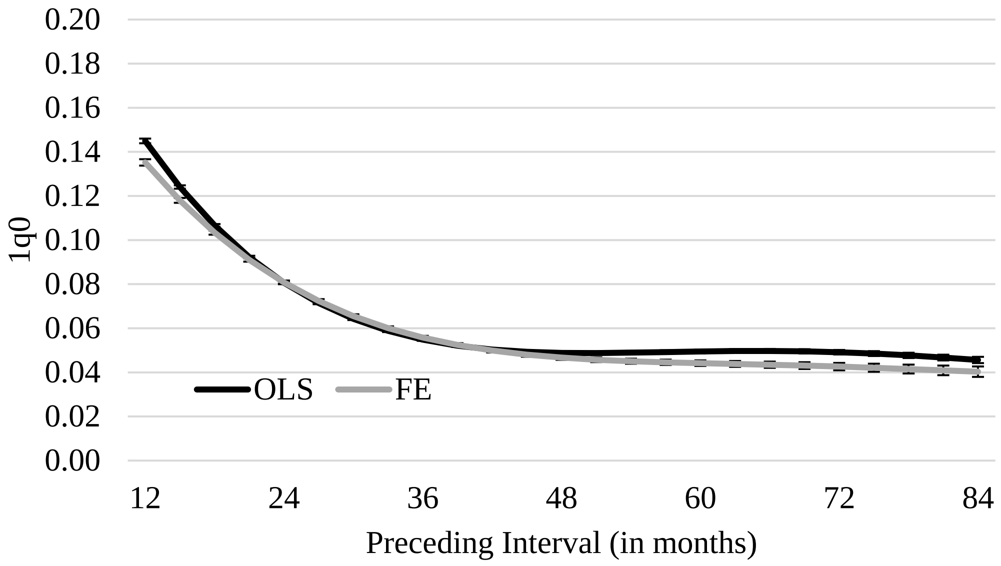

Figure A7. Predicted probabilities of dying before age 1 at different interval lengths in OLS and FE models among children born within ten years of survey.

Note: A ten-year cutoff was chosen because it has been shown that the misreporting and displacement of births tends to be low within that time frame (see Schoumaker 2014). Error bars represent 95% confidence intervals.

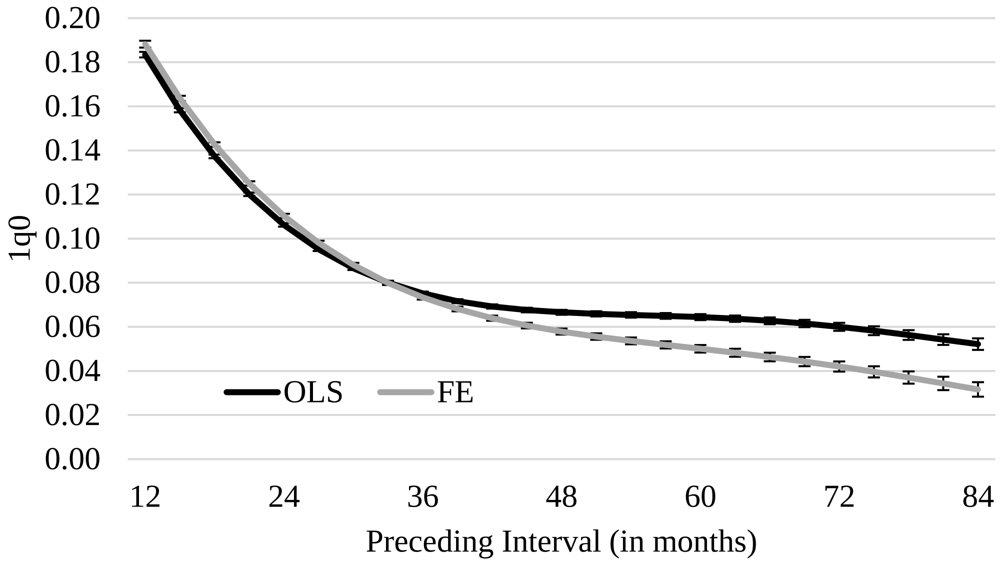

Figure A8. Predicted probabilities of dying before age 1 at different interval lengths in OLS and FE models among children born at even parities.

Note: Estimates based on models including only children born at even parities in families of four or more. This is to be sure that the death of the index child cannot also be included as the death of the previous sibling variable. Error bars represent 95% confidence intervals.

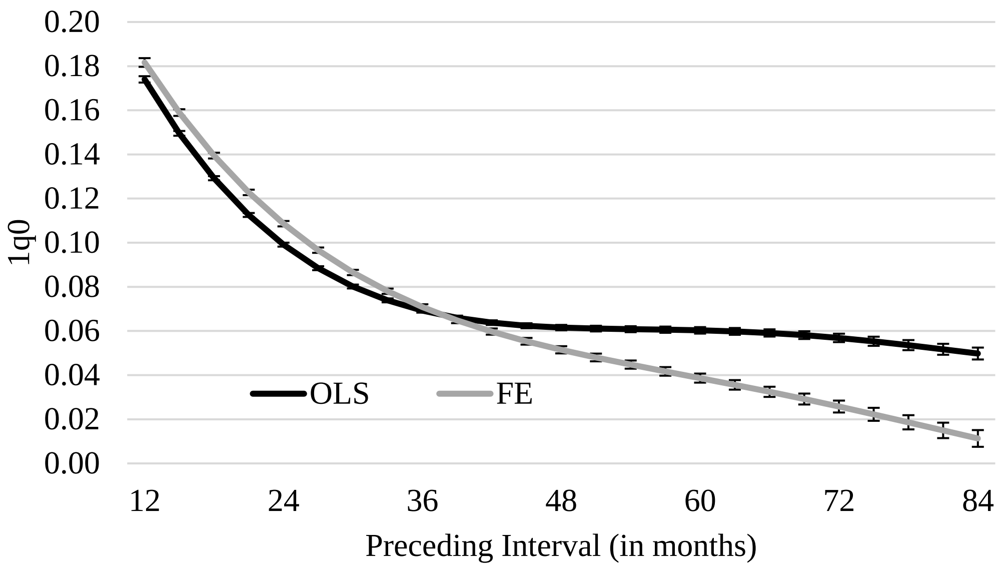

Figure A9. Predicted probabilities of dying before age 1 at different interval lengths in OLS and FE models among children born at odd parities.

Note: Estimates based on models including only children born at odd parities in families of five or more. This is to be sure that the death of the index child cannot also be included as the death of the previous sibling variable. Error bars represent 95% confidence intervals.

III. CHANGING REFERENCE INTERVAL LENGTH

(a) Infant Mortality Rate

(b) Total Fertility Rate

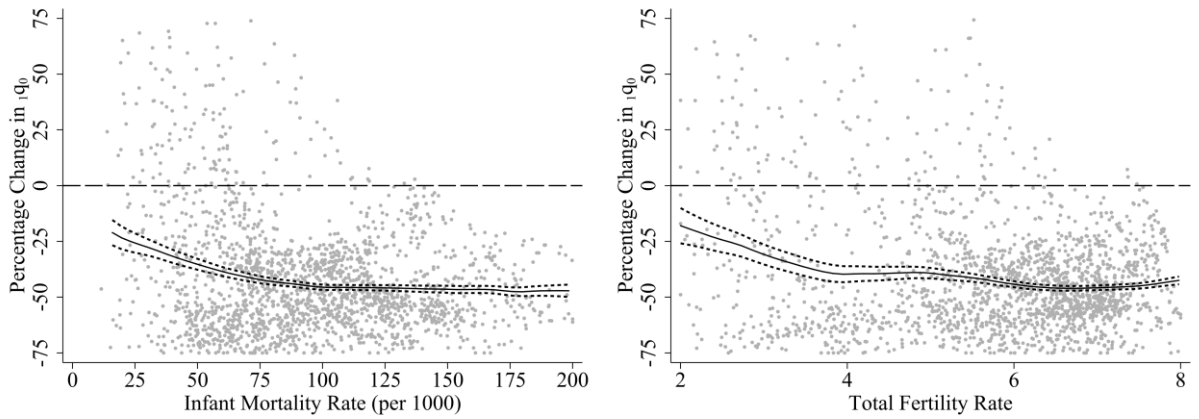

Figure A10. Marginal effect of increasing interval length from 18 to 30 months by the (a) infant mortality rate and (b) total fertility rate.

Note: A kernel-weighted local polynomial smoothed trend has been superimposed with 95% confidence intervals.

(a) Infant Mortality Rate (b) Total Fertility Rate

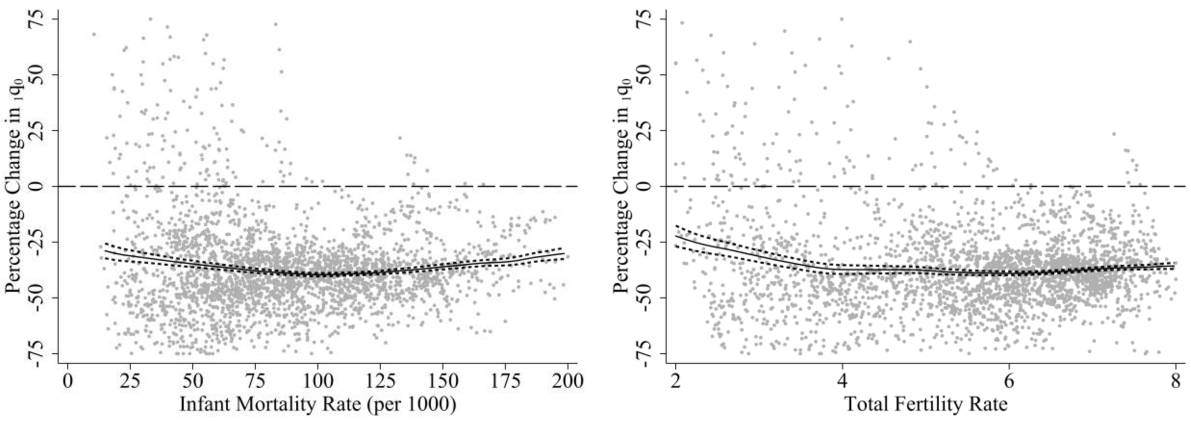

Figure A11. Marginal effect of increasing interval length from 24 to 36 months by the (a) infant mortality rate and (b) total fertility rate.

Note: A kernel-weighted local polynomial smoothed trend has been superimposed with 95% confidence intervals.

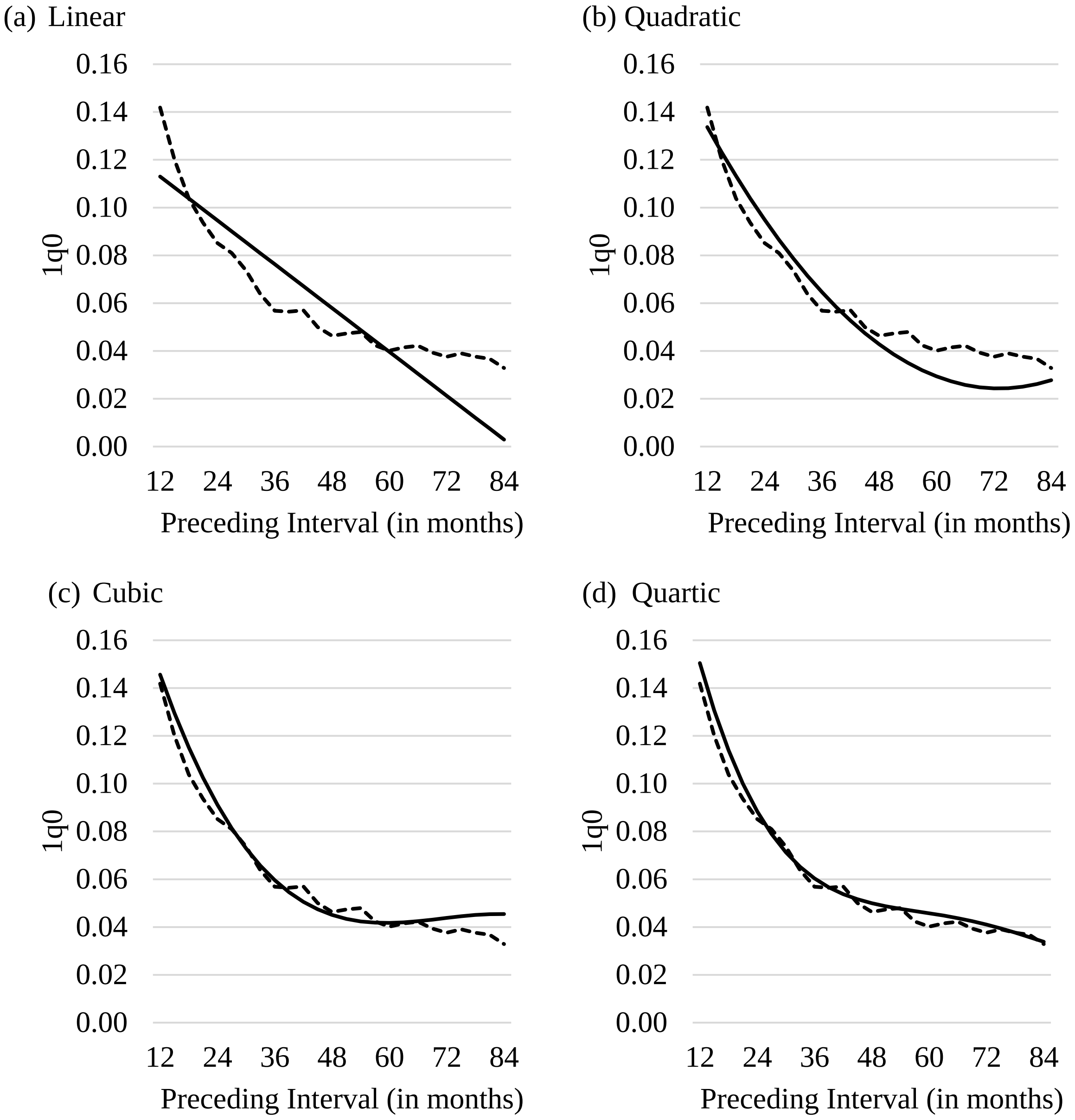

Figure A12. Comparing predicted probabilities of infant mortality from models using categorical and polynomial operationalizations of the length of the previous interval.

Note: Dashed line refers to the categorical operationalization.
